# Table of Tests and Solidities of the Alkaloids Appended

**Published:** 1880-10

**Authors:** 


Table of the Tests and Solibilities of the Alkaloids Appended.—By L. E. Sayre, Ph. G. George S. Davis, Medical Book Publisher, Detroit, Mich. 1880.

Transactions of the American Gynecological Society. vol. 4, for the year 1879. The revised press, Boston, 1880.

